# Endowing plants with a dual-cycle heart to boost CO_2_ fixation

**DOI:** 10.1093/nsr/nwag054

**Published:** 2026-01-28

**Authors:** Chunlei Zhao, Wenjie Hao, Xiulai Chen

**Affiliations:** Key Laboratory of Industrial Biotechnology of Ministry of Education, School of Biotechnology, Jiangnan University, China; Key Laboratory of Industrial Biotechnology of Ministry of Education, School of Biotechnology, Jiangnan University, China; Key Laboratory of Industrial Biotechnology of Ministry of Education, School of Biotechnology, Jiangnan University, China

**Figure 1.**
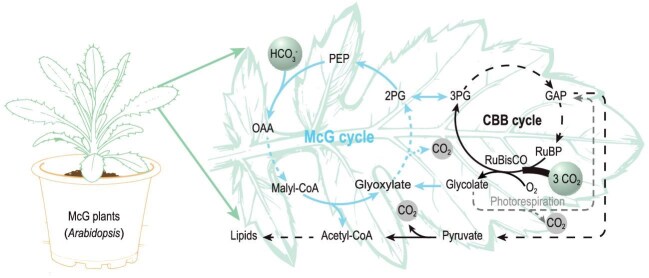
The CBB–McG dual cycle for carbon fixation. This dual-cycle system consists of the native CBB cycle and the synthetic McG cycle. This system begins with the carboxylation reaction of RuBisCO in the CBB cycle for fixing CO_2_ to produce 3PG, along with glycolate generation from the oxygenation reaction of RuBisCO. Subsequently, 3PG or glycolate enters the McG cycle, leading to the production of acetyl-CoA with one additional carbon fixation or without carbon loss. PEP, phosphoenolpyruvate; OAA, oxaloacetate; 2PG, 2-phosphoglycerate; RuBP, ribulose-1,5-bisphosphate.

Plant photosynthesis is a sustainable platform for addressing global food security and climate change [1]. However, the efficiency of photosynthesis for CO_2_ fixation is not economically efficient, primarily due to the inherent limitations of the Calvin–Benson–Bassham (CBB) cycle. Recently, synthetic carbon fixation pathways such as the malyl-CoA-glycerate (McG) cycle offer a promising alternative to mitigate these limitations in natural carbon fixation by improving carbon sequestration and emission reduction. Although these pathways have been designed and validated in microorganisms [2], their precise integration with the native metabolic network in higher plants remains a formidable challenge for successfully driving substantial growth advantages. A recent study published in *Science* by Lu and colleagues reports a groundbreaking effort in constructing a dual-cycle carbon fixation system by functionally integrating a synthetic McG cycle with the native CBB cycle in *Arabidopsis thaliana*, leading to a synergistic leap in biomass, leaf number, seed yield and lipid synthesis (Fig. 1) [3].

To fully tap the potential of plant synthetic biology for enhancing photosynthesis, three primary challenges need to be addressed effectively. First, natural carbon fixation systems in plants suffer from the fundamental limitations of CO_2_ utilization, due to the intrinsic inefficiency of ribulose-1,5-bisphosphate carboxylase/oxygenase (RuBisCO) carboxylation. Second, the photorespiratory metabolism of the RuBisCO oxygenation product glycolate to glyceraldehyde-3-phosphate (GAP) is constrained by the limited carbon yield, resulting from the release of photosynthetically fixed carbon as CO_2_ [4]. Third, the glycolytic conversion of the RuBisCO carboxylation product 3-phosphoglycerate (3PG) to acetyl-CoA is intrinsically accompanied by CO_2_-releasing decarboxylation [5]. In this context, Lu and coworkers present a synthetic biology solution that integrates a synthetic McG cycle with the native CBB cycle in *A. thaliana*, thereby constructing a dual-cycle carbon fixation system. This system channels both a RuBisCO carboxylation product (3PG) and an oxygenation product (glycolate) into acetyl-CoA synthesis by fixing one additional carbon or avoiding carbon loss with phosphoenolpyruvate carboxylase.

The engineering foundation of this study is the successful assembly of a synthetic McG cycle in the chloroplasts of *A. thaliana*, facilitating its synergistic

interaction with the native CBB cycle to create a dual-cycle carbon fixation system. To achieve this, the authors introduced six heterologous enzymes to construct the complete McG cycle and adopted synthetic biology strategies, including fusion proteins and promoter engineering, to ensure efficient localization and biologically active expression of these components in the chloroplasts. The refined dual-cycle system in *A. thaliana* was demonstrated by enabling the simultaneous utilization of the RuBisCO carboxylation and oxygenation products for efficient acetyl-CoA synthesis, thereby achieving additional carbon fixation and avoiding carbon loss.

To elucidate the mechanisms responsible for the significantly enhanced growth of McG plants, the authors performed a series of physiological and genetic analyses. They first quantified phytohormone levels and identified a substantial increase in cytokinins, which effectively explained the enlargement of the shoot apical meristem and the increase of the rosette leaf number, thereby leading to a tripled fresh weight (FW) and dry weight. This enhanced metabolic capacity led to tripled seed yields without extending the life cycle, demonstrating a breakthrough in developmental constraints. Furthermore, to trace the source of additional carbon flux, they constructed partial McG cycles and deleted glycolate dehydrogenase. This critical experiment confirmed that the conversion of glycolate into acetyl-CoA plays a dominant role in growth enhancement.

To quantitatively assess the effect of the dual cycle on photosynthetic performance, the authors conducted comprehensive analyses of CO_2_ assimilation rates, proteomics and enzyme activities. They found that the net CO_2_ assimilation rate under atmospheric conditions was doubled in McG plants. Notably, proteomic and enzyme activity analyses confirmed that this enhancement was not due to the increased RuBisCO abundance or activity, but attributable to the novel McG cycle functioning through phosphoenolpyruvate carboxylase. Further characterization of the CO_2_ response curve revealed a significantly reduced CO_2_ compensation point, demonstrating that the dual cycle effectively minimizes carbon loss by recycling RuBisCO carboxylation and oxygenation products. Additionally, the operating efficiency of photosystem II and the abundance of key photosynthetic proteins showed a consistent increase, confirming that this metabolic rewiring enhanced the overall performance of the photosynthetic system.

To trace the metabolic fate of the substantially enriched acetyl-CoA pool, the authors conducted comprehensive lipidomic profiling. Lipid droplets abundantly accumulated in the rosette leaves of McG plants. Subsequent quantitative analyses revealed lipid reprogramming in leaves, including a notable increase in triacylglycerol (TAG) contents from 0.015 to 3.0 mg g^−1^ FW, and significant alterations in fatty acids such as oleic and linoleic acids. In addition, TAG content in mature seeds was also doubled in McG plants. These results indicated that the McG cycle provoked a metabolic shift for directing carbon flux into storage lipid biosynthesis across both vegetative and reproductive tissues.

This research represents a paradigmatic breakthrough in plant synthetic biology, which provides a universal design framework for developing a highly modular metabolic ‘plugin’ to boost CO_2_ fixation. The dual-cycle system developed by Lu and coworkers facilitates the fundamental reprogramming of central carbon metabolism, leading to coordinated advancements in growth, yield and lipid synthesis. Thus, the established ‘C2-centric’ metabolic paradigm not only provides a novel solution to mitigate inherent carbon loss in plant photosynthesis but also opens new avenues for systematically enhancing plant carbon fixation.

The remaining challenge is how to broaden this proof of concept from a model plant, *A. thaliana*, to staple crops such as rice and wheat. This will require in-depth exploration into synthetic biology tools to optimize plant photosynthesis to achieve a net carbon gain and keep energetic costs down, coordinate expression of multiple enzymes in chloroplasts without imposing an excessive metabolic burden, and finally obtain a high-yield phenotype that is genetically stable across generations. This advancement will be essential for developing a new generation of engineered crops that maintain high-yield traits as consistently as elite cultivars under actual field conditions, or potentially demonstrate even greater stress resistance.

The dual-cycle system offers a new paradigm for addressing the inherent limitations of plant carbon fixation. Future optimization may proceed along three dimensions. First, high-atom-economy CBB–McG pathways can be developed by replacing the decarboxylation step in the McG cycle with the carboxylation reaction in the tartronyl-CoA (TaCo) pathway [6], thereby directly boosting plant carbon fixation. Second, rational engineering of the photosystem seeks to improve light capture and electron transfer efficiency, thereby providing sufficient driving force for carbon fixation. Third, the downstream pathways of key metabolic nodes can be reprogrammed to create an effective carbon sink for flexibly outputting the products of carbon fixation. In the near future, synthetic dual-cycle systems with iterative optimization are expected to be implemented in staple crops, thereby enhancing their photosynthesis and crop productivity. Ultimately, these synthetically redesigned crops are suitable for establishing a resilient and sustainable food production system that offers transformative solutions to global climate change and food security challenges.
